# Lead Optimization in Discovery Drug Metabolism and Pharmacokinetics/Case study: The Hepatitis C Virus (HCV) Protease Inhibitor SCH 503034

**Published:** 2007-06-26

**Authors:** K.-C. Cheng, Walter A. Korfmacher, Ronald E. White, F. George Njoroge

**Affiliations:** 1 Department of Drug Metabolism and Pharmacokinetics; 2 Chemistry Department Schering-Plough Research Institute, 2015 Galloping Hill Road, Kenilworth, NJ 07033 U.S.A.

## Abstract

Lead optimization using drug metabolism and pharmacokinetics (DMPK) parameters has become one of the primary focuses of research organizations involved in drug discovery in the last decade. Using a combination of rapid *in vivo* and *in vitro* DMPK screening procedures on a large array of compounds during the lead optimization process has resulted in development of compounds that have acceptable DMPK properties. In this review, we present a general screening paradigm that is currently being used as part of drug discovery at Schering-Plough and we describe a case study using the Hepatitis C Virus (HCV) protease inhibitor program as an example. By using the DMPK optimization tools, a potent HCV protease inhibitor, SCH 503034, was selected for development as a candidate drug.

## Introduction

Lead optimization in a drug metabolism environment is a multifaceted operation. It typically involves the use of various *in vitro* and *in vivo* screens to assess the drug metabolism and pharmacokinetic (DMPK) properties of multiple compounds, as well as to provide an early check on the safety issues that can be assessed in a higher throughput manner [1–4]. This process involves interaction between DMPK scientists, biologists/pharmacologists and medicinal/physical chemists. The goal of the interaction is to find a molecule that has the desired biological activity as well as DMPK properties and a safety profile appropriate for the targeted therapeutic indication. In this paper, we provide an overview of the DMPK lead optimization process that is used to support drug discovery projects at Schering-Plough. In addition, we will demonstrate how the process was used in a particular program (HCV protease inhibitor) as a case study.

## Lead Generation as a Part of New Drug Discovery

Contemporary parallel and combinatorial chemical synthesis produces large arrays of compounds that are available for evaluation in new drug discovery. Furthermore, other improvements by structural chemists using a variety of tools, such as X-ray crystallography, structural modeling and ligand/substrate docking algorithms, and by molecular biologists developing high-throughput binding targets and cell-based activity assays provide drug discovery scientists with an unprecedented level of structural-based rational designs to guide the synthesis of new chemotypes as potential drug leads. Along with the advancement of chemistry and biology, new automated *in vitro* activity screening tools have become commercially available which can carry out complex, programmable and adaptable robotic operations to test hundred of thousands of compounds in a speedy and precise manner. As a result, these new forces have worked together to increase our ability to create new chemical entities (NCEs) that exhibit the targeted pharmacological activity.

## Drug Metabolism as a Part of New Drug Discovery

**Figure 1.**
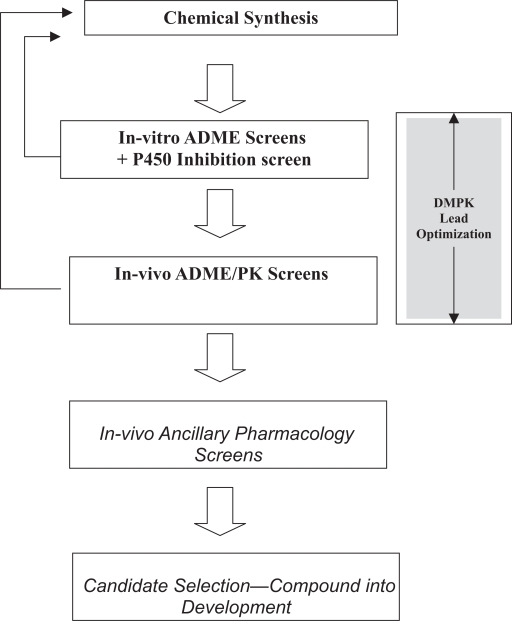
Scheme showing the iterative nature of lead optimization leading to candidate.

Several review articles in recent years have described the role that a drug metabolism and pharmacokinetics (DMPK) department can play in the process of new drug discovery [1, 2, 4–11]. As shown in Figure 1, DMPK provides the tools and the assays to assess various new chemical entities (NCEs) in terms of their absorption, distribution, metabolism and excretion (ADME) properties as well as their pharmacokinetic (PK) parameters. In addition, DMPK scientists may also use various screens to understand the potential of NCEs for preclinical or clinical toxicity [12]. The goal of these efforts is to find a compound that is suitable for development.

## Lead Optimization in a DMPK Environment

In order to understand the needs of lead optimization, it is important to define the basic characteristics of drug-like leads [7]. As shown in Table 1, there are at least five essential properties that need to be considered in order for a compound to be drug-like: potency, bioavailability, duration, safety and reasonable pharmaceutical properties. In addition, there are some other important properties, such as selectivity, efficacy and dose-proportionality, to be considered. A successful clinical drug candidate must at least meet the minimal acceptance criteria for each of these five properties for the type of drug program that is being developed. A major deficit in any one of the properties may prevent the compound from progressing from the drug development stage to the clinical phase or to the market.

During the discovery phase of lead optimization, the goal of the process is to find NCEs that fall into the acceptable range for each of these five properties. Among the five essential properties, three belong to the domain of DMPK: oral bioavailability, duration and safety issues. Hence, the lead optimization in discovery DMPK could be divided into three categories. First, for a drug to be given by oral administration (as is most often the case), the primary goal would be improving the oral bioavailability. This could be achieved by improving either the oral absorption or reducing the first-pass effect, or a combination of these. Secondly, improving the duration of the drug in the body could reduce the dose and the frequency of the dosing regimen. The duration of the drug in the body as measured by the half-life is inversely related to the systemic clearance of the compound. Therefore, improving (reducing) the systemic clearance of a series of compounds should extend their *in vivo* half-lives. Lastly, reducing any DMPK-related toxicity involves the use of multiple tests. For example, various tests are used in order to evaluate the potential for drug-drug interactions due to inhibition or induction of major CYPs, such as 3A4, 2D6, 1A2, 2C8, and 2C9. Another goal is to minimize the generation of reactive metabolites that may cause covalent binding. However, it is not totally clear whether the covalent binding may elicit any significant toxicity.

Many well-established assays/screens are now available for lead optimization in the DMPK environment (Table 2). These screens include both *in vitro* and *in vivo* assays. In the sections below, a more detailed discussion will focus on how many of these tools can be used in the lead optimization stage of new drug discovery.

## Improving Oral Bioavailability

Oral bioavailability (F) is governed by the absorption in the gastrointestinal (GI) tract and the fraction of the dose that is not metabolized by the GI tract or the liver (the first-pass effect) before it enters the systemic circulation. In a report by Chatuverdi et al. [1], oral bioavailability was defined as:

F = Fa ⋅ Fg ⋅ Fh ⋅ Fl

Fa is defined as the fraction of the drug that is absorbed across the intestinal wall, while Fg, Fh and Fl represent the fraction of the dosed drug that gets through the GI tract, the liver and the lung, respectively. A combination of *in vitro* and *in vivo* screens may be employed to assess preclinical absorption and used to predict human absorption.

The *in vitro* approach typically relies on using the Caco-2 system for screening the permeability of the NCEs. In addition to the caco-2 system, other types of membrane preparations or artificial membranes, such as isolated intestinal membrane and PAMPA, may be also suitable for the screening of permeability. Numerous reports have documented the utility of Caco-2 screening as well as the correlation between the Caco-2 permeability and the absorption in humans [13–16]. The Caco-2 system appears to be most predictable for compounds that are absorbed by the transcellular mechanism. Due to the small pore size of the tight junction, the Caco-2 system is less permeable to compounds that are absorbed by the paracellular mechanism. However, treatment of the Caco-2 cells with calcium-chelating reagents, such as EDTA, can increase the pore size of the tight junctions. This approach has been used to understand the potential paracellular permeability of lead compounds [17]. One of the drawbacks of using the caco-2 system is that the passive permeability may be underestimated for p-glycoprotein (p-GP) substrates due to efflux. The alternative is to use the PAMPA system which utilizes an artificial membrane, for the evaluation of passive permeability across membrane.

The *in vivo* approach to measure absorption in the discovery phase relies on animal pharmacokinetics. For instance, if the absorption of the lead compound is within the acceptable range in rodent and non-rodent species, such as dogs and monkeys, it is likely that human absorption may be within the acceptable range as well. To support this hypothesis, several publications [18, 19] have suggested that there is a correlation between the animal and human absorption, despite the fact that some distinct differences, such as the transit time, exist in the GI physiology between species.

The second element involved in the oral bioavailability is the first-pass effect. A compound entering the systematic circulation from the GI tract needs to first pass through two barriers—intestinal wall and the liver (this is often called the “first-pass effect”). Both the intestinal mucosa and the liver are enriched in drug metabolism enzymes. It has been well accepted that, due to species differences, animal metabolism may not be suitable for predicting the first-pass effect in humans. One common screen for estimating the human first-pass effect is to use microsomal preparations from human livers. The extraction ratio calculated from how quickly the NCE disappears in the microsomal incubation, may be used to estimate the extent of the liver first pass. With improving cryopreservation technologies, human hepatocytes have become a very useful tool in evaluating the metabolic clearance of test articles. A major advantage of using hepatocytes is that they contain both phase I and phase II metabolic enzymes. A fairly interesting approach to estimate the oral bioavailability was recently presented by using a Caco-2/hepatocyte hybrid system [20]. This novel system combines the Caco-2 permeability assay and the liver first pass assay into one system. As shown before [20], the Caco-2/hepatocyte system could provide a reasonable prediction of the oral bioavailability in humans. This approach could be used in conjunction with animal pharmacokinetic evaluation in lead optimization.

## Optimizing the Half-life of a Compound Series

The half-life of a compound is determined by both the clearance and the volume of distribution. As the clearance increases, the half-life decreases. Conversely, for a given clearance, a higher volume of distribution results in a longer half-life. For orally administered compounds, the apparent half-life is a combination of the elimination half-life and/or the absorption half-life. Hence, it is sometimes possible to develop a slow release formulation to extend the apparent half-life of a compound. However, the primary goal in the drug discovery phase is to optimize the half-life of the series of compounds. Frequently, the goal is to increase the half-life, while in certain cases shortening the half-life is the goal; half-life increase is generally done by trying to reduce the clearance of a compound. Since there are several ways to predict human clearance, the screening assays are designed according to these approaches. For example, allometry using animal clearance data has been often used to estimate the human clearance. This approach requires one to measure a compound’s clearance in at least three animal species and to use the animal clearances for allometric scaling. Therefore, using allometry to predict human clearance is hardly a high-throughput process.

In the lead optimization process, some of the most frequently used high-throughput in-vitro screens aim to predict hepatic clearance. These assays use either hepatic microsomes or primary hepatocytes derived from humans or animals. The general screening procedure monitors the disappearance of the NCE in an incubation mixture containing the compound and a fixed amount of microsomes or hepatocytes [1, 10, 21–23]. If the disappearance of the test compound follows first order kinetics, the rate of the process can be used to calculate the intrinsic clearance. A number of recent publications have suggested that using pooled hepatocytes from human donors results in a reasonable correlation between the measured intrinsic clearance and the in vivo clearance for a number of marketed compounds [24]. The predictive value of the hepatocyte clearance was demonstrated by a very good correlation (R^2^ = 0.86) when comparing the hepatocyte intrinsic clearance with *in vivo* clearance. Hence, by reducing the intrinsic clearance in an *in vitro* hepatocyte assay may predict improvement of the half-life.

## Safety/Toxicity Screening

Several potential safety issues can be related to DMPK properties. For example, toxicity due to drug-drug interactions that result from CYP isozyme inhibition or induction may cause a candidate drug to fail in development. In order to avoid these problems, NCEs are usually screened for their ability to inhibit major human CYP isozymes using either pooled human microsomes or supersomes which contain individual isozymes [17, 25–29]. It is also important to differentiate whether the observed inhibition is direct, metabolism or mechanism-based. The difference between the direct and the metabolism-based inhibition is that the direct inhibition by the parent compound is reversible whereas the metabolism-based inhibition is that a metabolite is a reversible inhibitor. A mechanism-based inhibition occurs when a reactive intermediate covalently modifies the CYP enzyme. The mechanism-based inhibition is usually irreversible. Practically, NCEs in drug discovery may encounter frequent CYP inhibition issues due to the wide substrate specificities of major human CYP isozmes, such as 3A4, 2D6, and 2C families.

CYP induction may cause opposite effects of CYP inhibition in that the exposure of the drug may be reduced. In rodents, the major induction issue appears to be the induction of the CYP 1A, CYP 2B, and CYP 3A family. There are considerably literatures suggesting that nuclear receptors, such as AhR, CAR and PXR, are involved in the induction of the respective CYPs [30]. In humans, the major induction pathway appears to be controlled by PXR. Administration of PXR agonists, such as rifampicin, causes elevated levels of intestinal and hepatic CYP 3A4, resulting in the reduction of oral bioavailability. CYP induction potential can be measured by certain *in vitro* assays, such as PXR-reporter gene assay and hepatocyte induction assay [4, 31].

## Human PK Prediction

The human PK and dose regimen prediction is also performed in order to facilitate the design and implementation of a clinical program. An evolving paradigm of human PK prediction combines allometry and scaling of the in vitro hepatic clearance. It is generally accepted that allometry using animal PK data may more accurately predict the volume of distribution and renal clearance, while it may be less accurate in predicting the hepatic clearance, since the metabolic enzymes, especially CYP enzymes, show significant differences between animals and humans. Hence, the use of intrinsic clearance obtained by microsomal or hepatocyte clearance assay for the scaling of in vivo clearance serves as an alternative way to predict the human hepatic clearance. When projected clearance values from allometry and in vitro-in vivo scaling are in reasonable agreement, one can feel confident that preclinical predictions of dosing regimen will not be highly inaccurate.

## Case study: HCV Protease Screening Paradigm

### Hepatitis C virus

Hepatis C virus (HCV), the etiologic agent of non-A, non-B hepatitis, represents a world wide-health problem, with approximately 170 million people infected with the virus [32]. Infection with HCV often leads to a chronic form of hepatitis. Without therapeutic intervention it could lead to cirrhosis, hepatic failure or hepatocellular carcinoma [33]. The current therapy for chronic HCV infection is subcutaneous injection of pegylated-interferon α alone or in combination with oral ribavirin [34]. HCV belongs to the family of *flaviviridae*, which includes other human pathogens, such as Yellow Fever and West Nile Virus. It is an enveloped positive stranded RNA virus. Upon entering a suitable host cell, the HCV genome serves as a template for cap-independent translation through its 5′ internal ribosome entry sites. The resulting 3000 amino acid polypeptide undergoes both co- and post-translational proteolytic maturation by host and virus-encoded proteases [35]. The virus-encoded protease responsible for processing the non-structural (NS) portion of the polypeptide is located in the N-terminal region of the NS3 protein. The NS3 protease structure provided necessary details to permit rational structure-assisted inhibitor design. This endeavor targeting the enzyme-substrate binding site resulted in the discovery of SCH 503034, a structurally novel ketoamide protease inhibitor. Recent proof-of-concept clinical studies with SCH 503034 and other HCV protease inhibitors BILN-2061 [36] and VX-950 [37] demonstrated the feasibility of targeting the protease.

### DMPK screening paradigm

**Figure 2.**
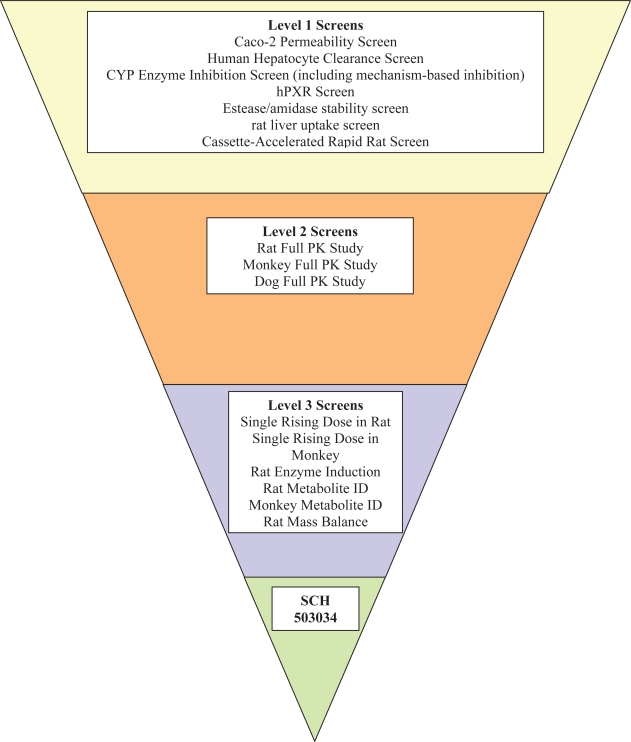
DMPK screening paradigm as part of the lead optimization and candidate selection process—application to HCV compound selection.

The following is a brief summary of the drug metabolism/pharmacokinetics process involved in the discovery of SCH 503034. About 10,000 compounds were synthesized and went through the cell-based assay (Replicon assay) for HCV protease inhibition activity. More than 1,000 compounds met the cut-off criterion of an IC90 of 1 μM or lower. These compounds were further optimized by DMPK (Fig. 2). Within the DMPK screening, several tiers were employed. In the first level, several higher throughput screenings were deployed: cassette-accelerated rapid rat screen (CARRS) screening [38], human hepatocyte clearance, Caco-2 permeability screening, and CYP enzyme inhibition (including mechanism-based inhibition) screening. In addition, some special screenings are also employed: plasma esterase/amidase screening and liver uptake screening. In the second level screening, more labor intensive assays were employed, such as full pharmacokinetic (PK) studies in rodent species and non-rodent species (monkey and dog). Of the 1000 compounds tested, three emerged as advance leads including SCH 503034. They appeared to meet the following acceptable DMPK criteria: moderate oral bioavailability in rats and dogs, absence of reactive metabolite, IC50 > 5 uM for CYPs 3A4, 2D6, 2C8, and 2C9, moderate huan hepatocyte clearance, and no CYP induction liability.

Following the DMPK screening process, a few advanced leads were identified that had acceptable DMPK characteristics. These advanced leads went through a DMPK profiling process for the final selection of the best compound for drug development (Level 3). These processes involved, for example, single rising dose studies in both rodent and non-rodent species to determine if desirable exposure multiples could be reached in the pre-clinical toxicology program. Then, multiple dosing in rats was performed in order to determine whether the circulating compound accumulates or produces auto-induction. Accumulation of the compound may indicate that the elimination of the compound, whether is due to hepatic clearance or renal clearance, or both, has been hampered.

**Figure 3.**
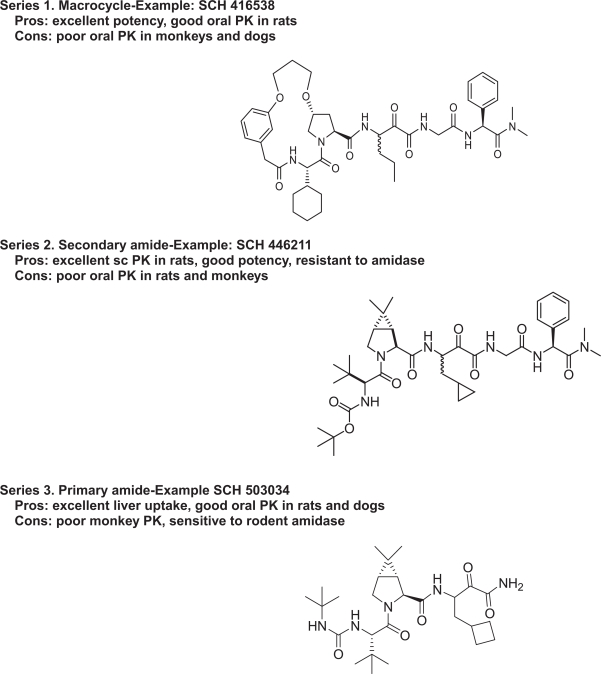
HCV compounds leading to SCH 503034.

Figure 3 shows the three chemotypes that were submitted for lead optimization in DMPK. A multitude of factors were involved in selection or elimination of certain chemotypes. A brief description of the DMPK properties of each of the chemotypes is discussed below:

### Macrocyclic compounds

An example compound from this series is SCH 416538 (Fig. 3). This type of compound showed resistance to peptidases and amidases. Since a more rigid conformation is maintained, this chemotype appears to have better potency. Some compounds in this class had good oral bioavailability in rats. However, the PK in dogs and monkeys was poor for most of the compounds.

### Secondary amides

An example compound from this series is SCH 446211 (Fig. 3). This chemotype showed reasonable half-life and excellent bioavailability after sub-cutaneous (SC) dosing, suggesting a potential dosing regimen through the SC route. Another advantage of this chemotype was the resistance to peptidases and amidases. Again, however, the oral bioavailability for this chemotype was very poor in rats and monkeys.

### Primary ketoamides

This class of compounds appeared to be very sensitive to rodent amidase. They were generally more resistant to human and non-rodent amidases in the plasma. Certain compounds in this class had good oral bioavailability in rats and dogs, but oral bioavailability in monkeys was poor. The major advantage of this chemotype is that liver uptake was found to be excellent. In addition, intrinsic clearance in human hepatocyte was acceptable. One compound in this series, SCH 503034, met the acceptance criteria for this program and was advanced into development.

## Conclusion

Higher-throughput DMPK screens using multiple *in vitro* and *in vivo* techniques are now in place and have become an essential part of the lead optimization process in new drug discovery. Future improvements in this lead optimization arena may be achieved by using automated systems to enhance the speed of these in-vitro screens. There is a continuing need in the area to improve the ability to predict *in vivo* pharmacokinetics by using *in vitro* evaluations of oral absorption, intestinal and hepatic first pass, hepatic intrinsic clearance, organ uptake and efflux mediated by transporters as well as plasma and cellular protein binding. In the near term, we will continue to use the combination of *in vitro* systems and fast *in vivo* screening for the selection of early discovery leads. In addition, *in vivo* studies for evaluation of the exposure multiple and metabolism and disposition may be accelerated in order to reduce the time to final candidate selection. Ultimately, it may become possible to use pharmacologically based *in silico* DMPK computer model parameters to support the rapid screening of drug candidates in order to shorten the time-frame of the lead optimization process while still discovering candidate drugs with acceptable DMPK properties.

## Figures and Tables

**Table 1.** General properties of drug-like lead compounds.

**Property**	**Definition/Requirement**
Potency	The intrinsic ability of a compound to produce a desirable pharmacological response (usually measured via high throughput in vitro screens)
Oral Bioavailability	The ability of a compound to pass through multiples barriers, such as the GI tract and the liver in order to reach the target
Duration (Half-life)	The ability of the compound to remain in circulation (or at the target site) for sufficient time to provide a meaningful pharmacological response
Safety	The compound has sufficient selectivity for the targeted response relative to non-targeted responses so that an adequate therapeutic index exists
Pharmaceutical Acceptability	The compound has suitable pharmaceutical properties, such as a reasonable synthetic pathway, adequate aqueous solubility, reasonable rate of dissolution, good chemical stability, etc.

**Table 2.** In vitro and In vivo DMPK screening tools.

**Assay Type**	**Assay**	**Species Relevance**	**References**
In-vitro	Caco-2	Human	[13–16]
In-vitro	Plasma Protein binding	Multiple	[39]
In-vitro	Intrinsic Clearance (microsomes or hepatocytes)	Multiple	[20–23]
In-vitro	CYP P450 Inhibition	Human	[25–29]
In-vitro	CYP P450 Induction	Human	[30–31]
In-vitro	CYP P450 Profiling	Human	[40]
In-vitro	Metabolite Profiling (microsomes or hepatocytes)	Multiple	
In-vitro	Transporter profiling	Human	
In-vivo	Rapid rat PK (CARRS)	Rat	
In-vivo	Single dose PK	Rat, Dog, Monkey	
In-vivo	Single rising dose PK	Rat and Dog or Monkey	
In-vivo	Metabolite Identification	Rat and Dog or Monkey	
In-vivo	Rat Mass Balance	Rat	
In-vivo	Multiple Rising Dose	Rat	
